# Retinoblastoma tumor suppressor pathway in breast cancer: prognosis, precision medicine, and therapeutic interventions

**DOI:** 10.1186/bcr3652

**Published:** 2014-05-07

**Authors:** Agnieszka K Witkiewicz, Erik S Knudsen

**Affiliations:** 1 Department of Pathology, Simmons Cancer Center, University of Texas Southwestern Medical Center, 5323 Harry Hines Blvd, NB6.416, Dallas, TX 75390-9072, USA

## Abstract

A series of recent studies have demonstrated that the retinoblastoma tumor suppressor (RB) pathway plays a critical role in multiple clinically relevant aspects of breast cancer biology, spanning early stage lesions to targeted treatment of metastatic disease. In ductal carcinoma *in situ*, multiple groups have shown that dysregulation of the RB pathway is critically associated with recurrence and disease progression. Functional models have similarly illustrated key roles for RB in regulating epithelial–mesenchymal transition and other features contributing to aggressive disease. Invasive breast cancers are treated in distinct fashions, and heterogeneity within the RB pathway relates to prognosis and response to commonly used therapeutics. Luminal B breast cancers that have a poor prognosis amongst estrogen receptor-positive disease are defined based on the expression of RB-regulated genes. Such findings have led to clinical interventions that directly target the RB pathway through CDK4/6 inhibition which have promise in both estrogen receptor-positive and Her2-positive disease. In contrast, RB loss results in improved response to chemotherapy in triple-negative breast cancer, where ongoing research is attempting to define intrinsic vulnerabilities for targeted intervention. These findings support a wide-reaching impact of the RB pathway on disease that could be harnessed for improved clinical interventions.

## Background

The retinoblastoma tumor suppressor (RB) is a potent regulator of cellular proliferation whose status provides critical information related to breast cancer prognosis and therapeutic interventions.

Although initially identified in a pediatric eye tumor, the last 30 years of research have demonstrated that RB plays an important role in many cancers. Loss of heterozygosity at the Rb1 locus represents the seminal basis for the development of retinoblastoma, and was the basis through which the gene encoding RB was identified [1-3]. RB has no known enzymatic activities, but functions in a host of processes by mediating protein interactions that are important for multiple phenotypes [4-7]. It is well known that RB binds to the E2F family of transcription factors and can repress the activity of E2F, leading to the attenuation of many genes that are required for cell cycle progression. This is very much the canonical view of RB in cell cycle control, although one should note that RB exerts many important affects that are independent/complementary. For example, RB can control chromatin cohesion, chromatin structure, tumorigenic proliferation, differentiation, and cell death through mechanisms beyond the dogmatic influence on E2F activity [4,7,8].

**Figure 1:**
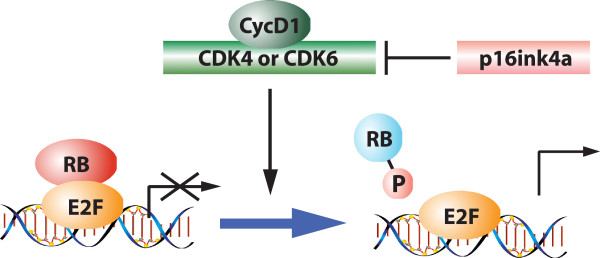
**Schematic of the retinoblastoma tumor suppressor pathway.** Diverse mitogenic and oncogenic signals induce the expression of D-type cyclins that activate CDK4/6. The resultant kinase activity is balanced by the CDK4/6 inhibitor p16ink4a. Typically, p16ink4a is at low levels in cells but can be induced by oncogenic or DNA damage stresses to suppress CDK4/6 activity. These signals coalesce to regulate the phosphorylation of the retinoblastoma tumor suppressor (RB). When active (unphosphorylated/hypophosphorylated), RB represses the activity of the E2F family of transcription factors and limits the expression of a program of genes required for S-phase (for example, MCM7 and Cdc6) and G2/M progression (for example, Cdk1 and cyclin B_1_). Phosphorylation relieves this transcriptional repression and allows for cell cycle progression. CDK, cyclin-dependent kinase; P, phosphorylated.

**Figure 2:**
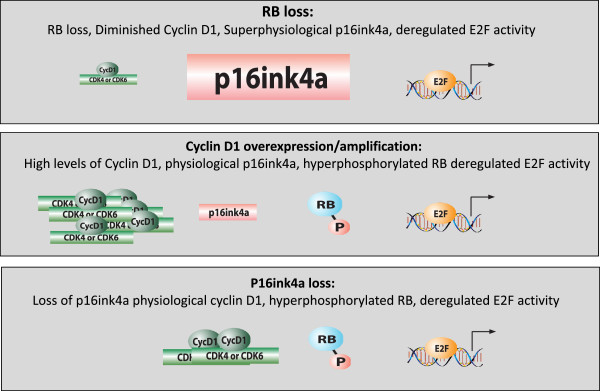
**Reciprocal inactivation of the retinoblastoma tumor suppressor pathway in cancer.** There are three primary mechanisms through which the retinoblastoma tumor suppressor (RB) pathway is inactivated in cancers that exhibit signature features. **(Top)** RB loss by genetic or epigenetic mechanisms results in the loss of the RB protein that is typically accompanied by exceedingly high levels of p16ink4a and relatively low levels of cyclin D_1_. **(Middle)** Cyclin D_1_ or CDK4/6 overexpression amplification is associated with intact RB and the relatively low levels of p16ink4a observed in normal tissue. **(Bottom)** Loss of p16ink4a is associated with intact RB and conventional levels of cyclin D_1_, as would be observed in a proliferative tissue. CDK, cyclin-dependent kinase; P, phosphorylated.

In spite of this complexity, there is wide agreement that RB must be inactivated for cell cycle progression, and thereby cell division, to occur. In normal physiology this is achieved via the phosphorylation of RB, which is catalyzed by cyclin-dependent kinases (CDKs) [9-11]. In particular, CDK4 and CDK6 are activated in response to the accumulation of D-type cyclins by mitogenic signaling and initiate the phosphorylation of RB [9,12,13]. CDK2 also plays a role in the phospho-inactivation of RB [14,15]. The biochemical importance of these processes was demonstrated using mutants of RB that cannot be phosphorylated and are potent inhibitors of cell cycle progression in the vast majority of tumor cells [10,11,16]. Furthermore, the CDK4/6 inhibitor p16ink4a is dependent on RB for function in suppressing cell cycle progression and inducing senescence [17-20]. Together, the pathway of CDK4/6, RB, and p16ink4a define the proximal RB pathway (Figure 1). Importantly, this pathway demonstrates mutually exclusive disruption in cancer [21-24]. For example, tumors with loss of p16ink4a will retain wild-type RB, while tumors mutant for RB will express p16ink4a at very high levels (Figure 2).

Although the RB pathway is often simplified to the level that all perturbations are viewed as being equivalent, there are clearly unique features of CDK4/6, p16ink4a, and RB biology. For example, CDK4/6 and cyclin D_1_ have been shown to have important targets beyond the phosphorylation of RB [25-27]. p16ink4a and RB are members of gene families, and compensatory mechanisms can mitigate the effects of loss of these tumor suppressors in specific settings, most notably in mouse models [28-30]. Additionally, phosphorylation of RB can be regulated by a plethora of mechanisms beyond CDK4/6 [9,31] and, due to its downstream position in the pathway, RB loss has a particularly significant effect on cell cycle control.

The analysis of many tumor types has indicated that the RB pathway is perturbed in some form or another in most cancers. However, there is often a tumor-specific tropism for the mechanisms of pathway inactivation [21,23,24,32]. For instance, pancreatic cancers frequently lose p16ink4a, while osteosarcomas often lose RB. There is thus both common loss of the pathway and intrinsic diversity based on the mechanism through which the pathway is inactivated.

In breast cancer, different subtypes of disease are dominated by differential mechanisms of RB pathway inactivation. Estrogen receptor (ER)-positive breast cancers generally exhibit deregulation of the kinase components CDK4/6 as a result of aberrant cyclin D_1_ expression or amplification [33-37]. Her2-positive breast cancers typically push on the pathway through D-type cyclins, and similarly there are few cases that exhibit RB loss. In contrast, loss of the *Rb1* gene and the RB protein has been documented at high frequency in triple-negative breast cancer (TNBC) [38,39] . In spite of these generalities, one should note that any breast cancer can exhibit loss of RB, loss of p16ink4a, or amplification of cyclin D_1_; there is thus the opportunity to evaluate how these events impinge on the underlying biology of disease and the prognostic and therapeutic implications in the clinic.

## Retinoblastoma tumor suppressor pathway disruption in ductal carcinoma in situ

The majority of invasive breast cancers are believed to develop from precursor lesions. In particular, ductal carcinoma *in situ* (DCIS) is considered the precursor to the majority of breast cancers [40,41]. With standard use of mammography, the frequency of DCIS diagnosis has increased over 20-fold in the last 20 years [39]. The control rates for DCIS are very good and women with a DCIS diagnosis are generally treated with minimally invasive surgery (that is, lumpectomy) coupled with adjuvant radiation therapy [42,43]. However, it is apparent that most DCIS cases do not require radiation, and in fact most women are overtreated [40]. In a review of large clinical trials on the treatment of DCIS, the recurrence rate is approximately 30% with surgery alone but approximately 15% with the inclusion of radiation. This means radiation induces a significant clinical benefit. However, ~70% of the women who were treated with radiation would have not had their cancer return; they were therefore overtreated. In contrast, there are ~15% of women for whom an even more effective treatment is needed. For these reasons there has been a lot of interest in understanding determinants of recurrence and progression to invasive disease in DCIS.

**Figure 3:**
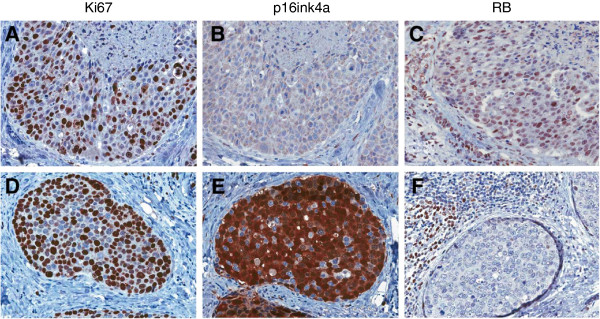
**Representative staining patterns observed in ductal carcinoma*in situ*. (A,B,C)** One case retains intact retinoblastoma tumor suppressor (RB) and the relatively low levels of p16ink4a as observed in most tissues. **(D,E,F)** The other case has lost RB and expresses very high levels of p16ink4a.

Early functional studies from Tlsty’s group and others suggested that the CDK4/6 inhibitor p16ink4a could be a particularly important factor in suppressing the progression of DCIS [44-46]. Such a model is consistent with the finding that high levels of p16ink4a represent a significant barrier to oncogenic conversion. For example, high levels of p16ink4a in benign Nevi are believed to contribute to potent suppression of melanoma [18]. Paradoxically, high levels of p16ink4a, particularly in conjunction with a high proliferation index, were associated with disease recurrence and progression [47]. Such a combination of markers (high p16ink4a and high proliferation) is indicative of the loss of RB. This is supported by a multitude of studies showing that p16ink4a levels are very high in tumors that have lost RB by mutation or through the action of viral oncoproteins [48]. Furthermore, only through the loss of RB can the cytostatic effect of p16ink4a be bypassed [17]. Subsequent work validated the primary findings in independent cohorts [49,50]. Importantly, subsequent direct analysis of RB loss in DCIS by optimized immunohistochemistry revealed that RB loss is one of the strongest markers of DCIS recurrence and progression that has been identified and does occur in tumors that express high levels of p16ink4a [51] (Figure 3). The prognostic significance of RB-pathway deregulation is significant in multivariate models, and is true both as a single marker and in combination with other determinants of DCIS biology, including Her2 levels, Cox2 levels, and PTEN levels [49-52].

Defining the mechanisms underlying the progression of DCIS has been the subject of recent intense study. Functionally, the transition between DCIS and invasive breast cancer represents invasion through ductal myoepithelium and basement membrane into the surrounding tissue. Molecular analysis comparing DCIS with invasive breast cancer demonstrated that one of the key differences between these disease states is the presence of epithelial–mesenchymal transition (EMT) in invasive cancer [53,54]. This finding emerged from independent groups using unbiased gene expression profiling on microdissected tissues. Interestingly, several groups have demonstrated that, in addition to its canonical effects on proliferation, RB loss can lead to EMT or a partial EMT [52,55,56]. Particularly in a variety of breast cancer models, knockdown of RB led to altered morphology and the expression of specific markers of EMT (for example, vimentin) [56]. These outcomes were ostensibly driven through the induction of the activity of EMT-mediators Zeb1 and Slug. Similarly, in mouse models it was observed that tumors which arise with Rb1 deletion are particularly characterized by aspects of EMT [57]. Lastly, in models of DCIS progression using three-dimensional cultures of MCF10A cells, RB deficiency drove an altered gene expression program indicative of loss of epithelial characteristics [52].

The results from these multiple lines of investigation support a critical role for RB in DCIS progression. Most probably, RB loss represents a particularly potent hit for DCIS as it both relieves control over the cell cycle allowing for expansion of DCIS cells and promotes EMT-like processes that promote invasion. These effects of RB loss clearly occur in the context of other oncogenic events (for example, loss of PTEN or Her2) that cooperate in the genesis of DCIS and its ultimate progression to invasive disease.

These provocative findings support the concept that RB pathway status could be utilized to direct the care of patients with DCIS. For example, cases of DCIS that are p16ink4a high and RB deficient would be expected to have the most benefit from adjuvant radiation therapy. In contrast, tumors that are RB proficient would have a low risk of recurrence/progression and therefore may not require radiation treatment. Unfortunately, in spite of the data published thus far, no analysis of clinical specimens has addressed whether the RB pathway status can be used specifically to direct the care of DCIS. Additional investigation is therefore required to bring the RB status to clinical application in DCIS.

### Multiple breast cancer subtypes necessitate a focus on subtypes as individual diseases

Unlike the one-size-fits-all approach to DCIS, invasive breast cancer represents at least three distinct diseases with differing prognosis and therapeutic interventions that are based on well-established molecular markers. The three major clinicopathologic subtypes of breast cancer (ER-positive, Her2-positive and triple-negative) are defined based on the presence of specific proteins in tumors that are therapeutically actionable. A plethora of molecular studies have served to refine these subtypes and provide added insight into the underlying biology of these forms of disease [58,59]. In each of these disease contexts, the RB pathway plays discrete roles that have significant clinical implications for the management of breast cancer.

## Estrogen receptor-positive breast cancer: the retinoblastoma tumor suppressor pathway as a prognostic tool and therapeutic target

**Figure 4:**
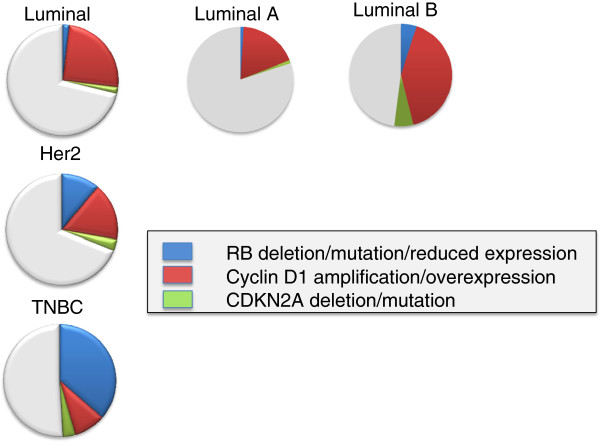
**The retinoblastoma tumor suppressor pathway in different breast cancer subtypes via TCGA.** CBIOPORTAL was used to access data from the TCGA. The canonical retinoblastoma tumor suppressor (RB) pathway was evaluated for both genetic aberrations and altered expression. TNBC, triple-negative breast cancer.

Constituting ~70% of diagnosed breast cancer, ER-positive breast cancer is the most prevalent form of breast cancer and is treated based on the presence of the ER. As mentioned above, ER-positive breast cancer is dominated by deregulation of CDK4/6 that is driven by amplication or overexpression of cyclin D_1_[36,60] (Figure 4). This event has been hypothesized to contribute to more aggressive forms of ER-positive breast cancer, although this hypothesis remains controversial.

### Prognostic retinoblastoma tumor suppressor/E2F signatures

**Figure 5:**
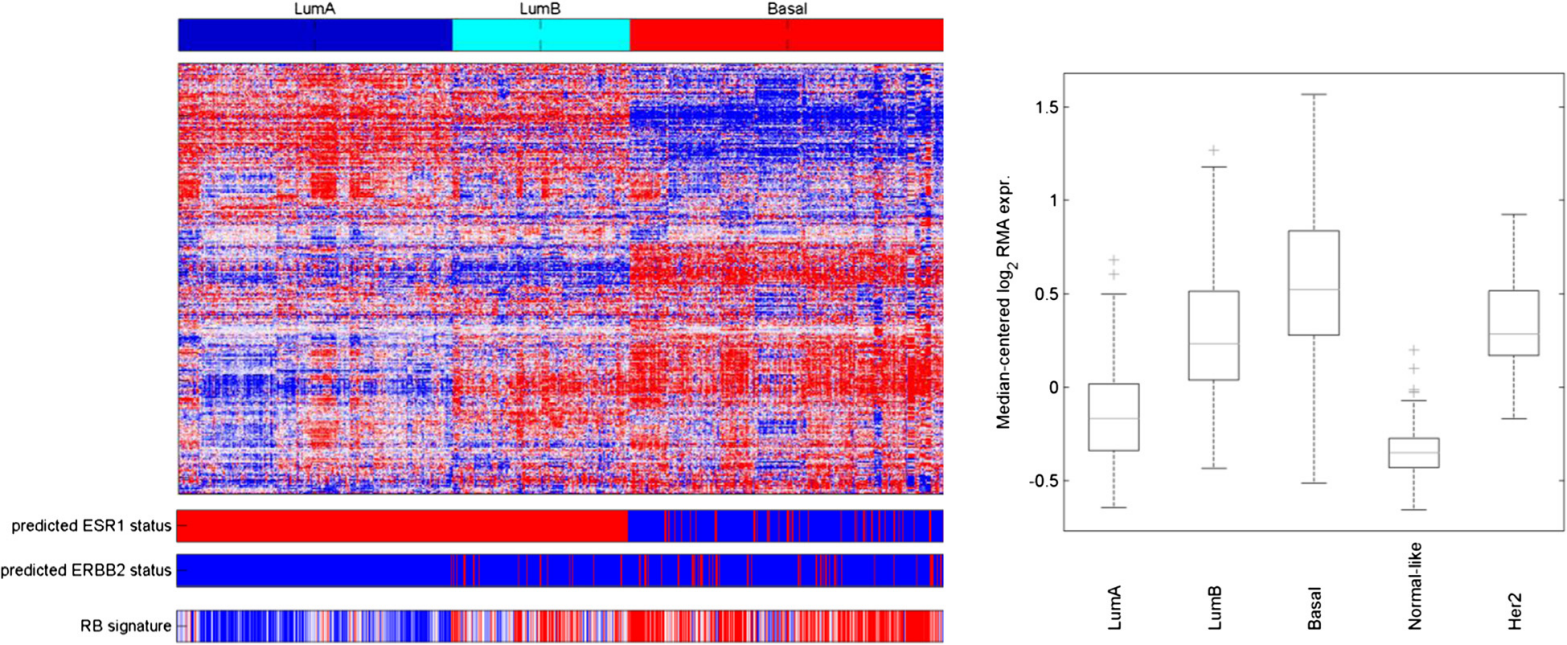
**Gene expression analysis of a retinoblastoma tumor suppressor/E2F signature in breast cancer. (Left)** The intrinsic subtypes of breast cancer were defined based on conventional gene expression properties. Luminal (Lum)B and basal cancers are characterized by elevated expression of retinoblastoma tumor suppressor (RB)/E2F-regulated genes (highlighted with bracket). The tumors exhibit deregulation of RB/E2F-regulated genes as shown by specific analysis of an RB signature. **(Right)** The relative level of the RB signature was evaluated across five breast cancer subtypes. Data demonstrate the high levels of pathway deregulation in luminal B, basal, and Her2-positive cancers that have intrinsic poor prognosis.

What is clearly without contention is that breast cancers which harbor deregulated expression of RB/E2F target genes are associated with poor prognosis [61-63]. In fact, the expression of the proliferation-associated RB/E2F target genes provides the basis for the distinction between luminal A and luminal B breast cancer [59]. An example is shown in Figure 5, where deregulation of an RB/E2F signature differentiates luminal A and luminal B breast cancer. Simple analysis of TCGA datasets indicates that luminal B cancers are over-represented for cyclin D_1_ amplification and loss of p16ink4a or RB relative to luminal A breast cancer (Figure 4). Deregulation of the RB pathway thus does seem to associate with more aggressive tumor behavior in ER-positive breast cancer. Consistent with this point, perturbation of the RB pathway has been shown to lead to more aggressive/rapid tumor growth in preclinical models [64].

One of the most significant challenges in the management of ER-positive breast cancer is relapsed disease. Unlike other forms of breast cancer, recurrence can occur very late after treatment, and suggests that deposits of ER-positive breast cancer can survive in a dormant state for multiple years. Luminal B tumors are known to exhibit rapid recurrence with endocrine therapy. Such tumors are routinely detected in the clinic through the use of existing diagnostic tests. For example, oncotypeDx estimates the risk of recurrence using a proprietary algorithm that is dependent on the expression of proliferation-associated genes that are controlled by RB/E2F [65,66]. Deregulation of RB/E2F activity is thus, in effect, used clinically to estimate the risk of recurrence. In the case of ER-positive breast cancer, this information is employed to direct adjuvant chemotherapy as a means to reduce the subsequent risk of recurrence.

### Impact of the retinoblastoma tumor suppressor pathway on therapeutic resistance

Knowledge that deregulation of the RB pathway is associated with rapid recurrence with endocrine therapy is born out in multiple studies. Initially, endocrine therapy was demonstrated to impinge on the RB pathway to elicit cell cycle inhibition [67]. Similarly, perturbation of the RB pathway through multiple mechanisms can blunt the sensitivity to endocrine therapies and can help facilitate the development of resistance [64,68]. While important and demonstrating that downstream aberration of the RB pathway can bypass the response to endocrine therapy, loss of RB or cyclin D_1_ amplification does not represent the principle basis for acquired resistance in preclinical models. Typically, deregulation of oncogenic signaling molecules will drive the aberrant activation of cyclin D_1_ and RB phosphorylation independent of ER [69]. More recently, it was shown that ER mutations which drive resistance to endocrine therapy are selected in metastatic disease. Therefore, while cyclin D_1_ and RB are important determinants of response, multiple paths exist that lead to the RB pathway and bypass of endocrine therapy.

### Direct targeting of the retinoblastoma tumor suppressor pathway in estrogen receptor-positive breast cancer

**Figure 6:**
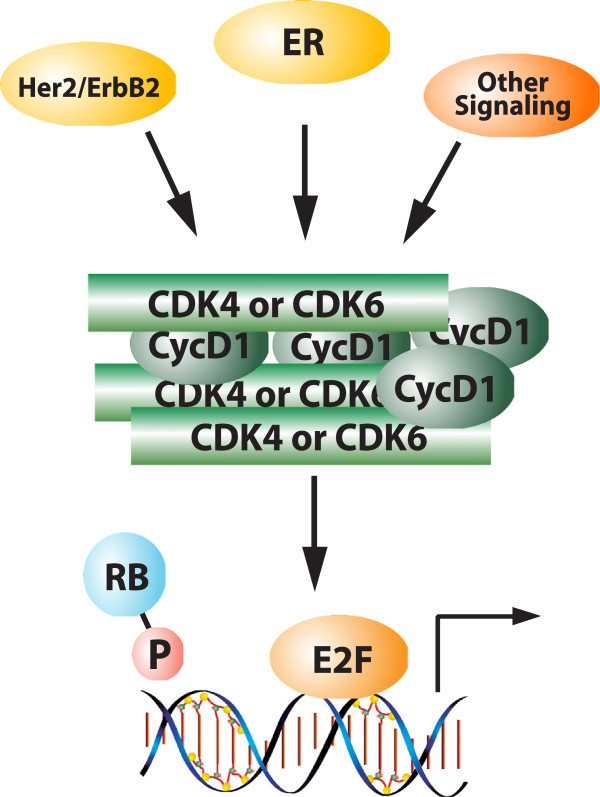
**CDK4/6 is downstream of multiple pathways.** Estrogen receptor (ER) and Her2 as well as many oncogenic mechanisms (for example, phosphatidylinositol 3-kinase) ultimately impinge on the activity of CDK4/6 to drive cell cycle progression. CDK, cyclin-dependent kinase; RB, retinoblastoma tumor suppressor; P, phosphorylated.

If the RB pathway is in fact relevant for ER-positive breast cancer, one would expect that interventions which act specifically upon RB would be relevant for disease control. Tumors that retain RB are dependent on CDK4/6 to phosphorylate the tumor suppressor for cell cycle progression. Several specific CDK4/6 inhibitors have been developed. These inhibitors are highly specific to RB-positive tumors and can induce profound cytostatic effects in spite of multiple oncogenic signals that are well known to bypass endocrine therapy [70,71]. Additionally, it has been shown that such agents are exceedingly effective at blocking the proliferation of models that are resistant to endocrine therapy, probably because CDK4/6 is downstream of multiple pathways (for example, Her2 and PI3K) that are implicated in the bypass of endocrine therapy [70,72,73] (Figure 6).

In considering the utility of such agents in the clinic, there are three key features to consider. First, such agents can act in concert with endocrine therapy, and through their cytostatic action can limit the selection for resistance to the primary endocrine agent [70]. Second, such agents can function to prevent the expansion of resistant clones that might emerge. Third, because the CDK4/6 inhibitor is independent of ER signaling, even models that are completely resistant to endocrine therapy would still exhibit benefit [70,72,73]. Based on these considerations, clinical trials have been initiated of CDK4/6 inhibitors in conjunction with endocrine therapy (Table 1). The most advanced of these trials involves the Pfizer CDK4/6 inhibitor (PD-0332991; Pfizer; New York, NY USA) in combination with the aromatase inhibitor letrozole for patients with recurrent disease. The interim data that have been presented for this trial demonstrate an incredible threefold enhancement of progression-free survival in a randomized phase 2 trial [74]. The findings from this work have spawned a plethora of clinical trials investigating CDK4/6 inhibitor breast cancer, and support the overall contention that the RB pathway does represent an actionable target in ER-positive breast cancer.

**Table 1:** Trials of CDK4/6 inhibitors in breast cancer

**Trial of CDK4/6 agent**	**Target disease**	**Trial phase**
**PD-0332991/Palbociclib (Pfizer; New York, NY, USA)**		
A Study of Palbociclib in Addition to Standard Endocrine Treatment in Hormone Receptor Positive Her2 Normal Patients with Residual Disease after Neoadjuvant Chemotherapy and Surgery (PENELOPE-B) (ClinicalTrials.gov NCT01864746)	ER-positive (adjuvant)	III
PD 0332991 and Anastrozole for Stage 2 or 3 Estrogen Receptor Positive and HER2 Negative Breast Cancer (ClinicalTrials.gov NCT01723774)	ER-positive (neoadjuvant)	II
A Study of PD-0332991 + Letrozole vs. Letrozole for 1st Line Treatment of Postmenopausal Women with ER^+^/HER2^-^ Advanced Breast Cancer (ClinicalTrials.gov NCT017240427)	ER-positive (first-line advanced)	III
PD0332991/Paclitaxel in Advanced Breast Cancer (ClinicalTrials.gov NCT01320592)	All metastatic	Ib
**LEE011 (Novartis; New York, NY, USA)**		
Phase Ib/II Trial of LEE011 with Everolimus (RAD001) and Exemestane in the Treatment of ER^+^Her2^-^ Advanced Breast Cancer	ER-positive (advanced/metastatic)	Ib/II
Study of LEE011, BYL719 and Letrozole in Advanced ER^+^ Breast Cancer (ClinicalTrials.gov NCT01872260)	ER-positive (advanced/metastatic)	Ib/II
A Pharmacodynamics Pre-surgical Study of LEE011 in Early Breast Cancer Patients (MONALEESA-1) (ClinicalTrials.gov NCT01919229)	ER-positive (neoadjuvant)	II
Study of Efficacy and Safety of LEE011 in Postmenopausal Women with Advanced Breast Cancer (MONALEESA-2) (ClinicalTrials.gov NCT01958021)	ER-positive (advanced/metastatic)	II

*CDK*, cyclin-dependent kinase; *ER*, estrogen receptor.

## Her2-positive breast cancer: an opportunity

Her2-positive breast cancer constitutes ~20% of diagnosed breast cancers. In general, Her2-positive disease is more aggressive than ER-positive disease, although the treatment paradigm has many parallels with ER-positive breast cancer. For example, Her2-positive disease is treated based on an addiction to Her2 signaling, and a plethora of active Her2-targeted agents are now employed in the clinic [75]. Like ER-positive disease, the principle clinical issue remains recurrence or intrinsic resistance to the front-line therapy. However, other than Her2 itself, there are no markers to direct treatment decisions, and there is no clear indication that the status of the RB pathway impinges on the sensitivity to Her2-targeted therapy. In spite of the relatively limited analysis of the RB pathway in clinical specimens, there are compelling reasons to believe that the pathway will be relevant as a therapeutic target in Her2-positive breast cancer.

## Preclinical data demonstrate a key dependence of Her2 disease on the retinoblastoma tumor suppressor pathway

Genetically engineered mouse models provide an important means to evaluate genetic dependencies in tumor development. Sicinski’s laboratory investigated the relationship of cyclin D_1_ genetic loss with the development of mammary tumors in mice whose tumors were driven by multiple different genetic stresses [76]. While Myc or Wnt oncogenes can effectively drive tumor development irrespective of cyclin D_1_, genetic deletion of cyclin D_1_ specifically prevented tumor development driven by Her2 [77]. More recently, a conditional knockout approach showed that cyclin D_1_ does represent a therapeutic target in Her2-positive breast cancer [78]. Parallel investigation has shown that Her2-positive models are sensitive to CDK4/6 inhibitors in preclinical models; interestingly, in such models CDK4/6 inhibition not only prevents proliferation but also limits the invasive potential of such models [52]. Therefore, in addition to preventing tumor growth, such an arrest compromises invasive potential. Presumably, as with ER-positive breast cancer, the efficacy of CDK4/6 inhibition in this context is due to its position downstream of the canonical Her2 signaling. Clinical trials are now being initiated to specifically address the ability of CDK4/6 inhibitors to add to the control of Her2-positive cancers.

## Triple-negative breast cancer

TNBC is widely accepted to represent the biggest clinical challenge in breast cancer [78,79]. TNBC is highly aggressive and almost certainly represents several different diseases that have different sensitivities to therapies. Currently, all patients with a TNBC diagnosis are treated with cytotoxic chemotherapy. Such therapy can be incredibly effective for a subset of patients (~30% ), while many patients do not effectively respond and will undergo rapid recurrence [80]. Unfortunately, there are minimal therapeutic interventions for such patients; thus, while TNBC constitutes a relatively minor fraction of breast cancer cases (~20% ), almost 50% of cancer-associated deaths are associated with TNBC. Additional targeted approaches are therefore urgently needed.

**Figure 7:**
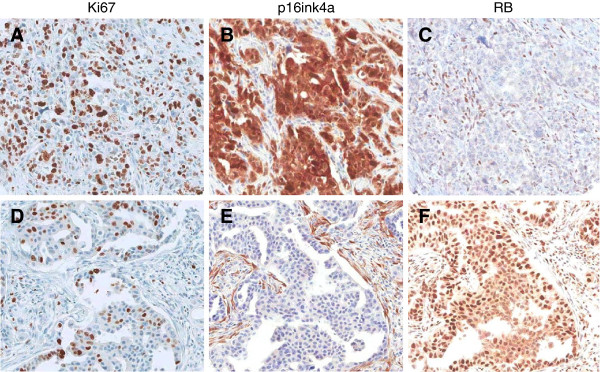
**Representative staining patterns observed in triple-negative breast cancer. (A,B,C)** One case retains intact retinoblastoma tumor suppressor (RB) and the relatively low levels of p16ink4a as observed in most tissues. **(D,E,F)** The other case has lost RB and expresses very high levels of p16ink4a.

Unlike Her2-positive or ER-positive breast cancers that have relatively limited loss of RB, TNBC exhibits frequent loss of RB as determined by histological analysis [38,39]. Such a finding is also supported by the high levels of p16ink4a observed in many TNBC cases [81]. Lastly, TNBC has very high levels of RB/E2F signature genes relative to other tumor subtypes [62,63]. Representative images of RB-positive and RB-deficient TNBC are shown in Figure 7. Gene expression analysis and immunohistochemical approaches have shown that tumors that lack RB have a good response to chemotherapy, as indicated by a pathological complete response in neoadjuvant studies or improved overall outcome [62,63,82]. This finding is counterintuitive because it suggests that the most aggressive rapidly growing tumors in fact have the best prognosis. The prevailing view of this paradox is that such rapidly proliferating tumors lack critical RB-mediated cell cycle checkpoints and are thus very sensitive to chemotherapy [61]. Such a concept is supported by preclinical data from multiple laboratories showing that RB loss is associated with sensitivity to chemotherapeutic agents. Recent drug screening efforts indicate a complex combination of responses that is conditioned by the therapeutic intervention employed [83]. If more extensively validated, these data would suggest that RB loss could be specifically used to define patients whose tumors would be most apt to respond to optimized chemotherapy regimens.

These findings from RB pathway-based analysis are largely consistent with recent findings from Pietenpol’s group that TNBC represents several intrinsic subtypes [84]. Those subtypes with the highest expression of RB/E2F-regulated genes are generally more sensitive to chemotherapy. In contrast, other subtypes can be treated with other agents. For example, the luminal AR subtype can be treated with androgen receptor antagonists [84]. What remains unclear is whether the analysis of RB/E2F does in fact define subtypes that have improved response to chemotherapy (for example, basal-like cancers) or whether it provides additional information to the subtypes that have been elucidated.

## Defining targeted treatments of triple-negative breast cancer through retinoblastoma tumor suppressor

Because RB is lost in ~40% of TNBC, one would expect that it could be used to define unique sensitivities upon which to direct treatment. Anecdotal clinical data suggest that CDK4/6 inhibition may not be particularly effective in TNBC. There are multiple possible explanations for this, including the fact that TNBC is inherently heterogeneous, and most probably single targeted agents will never have much impact on advanced disease that has failed prior chemotherapy. Approaches to define drugs that interact positively with CDK4/6 inhibitors are therefore incredibly important. Unlike endocrine therapy or Her2-targeted therapies that interact positively with CDK4/6 inhibitors, multiple laboratories have shown antagonism with chemotherapy [85,86]. As such, either metronomic approaches will be needed or it will be critical to define targeted agents that have positive interactions with CDK4/6 inhibitors in the treatment of TNBC.

While RB-deficient tumors do respond better to conventional chemotherapy, the unfortunate reality is that many RB-deficient tumors fail to respond or undergo recurrence. These findings have lead to the pursuit of drugs that exploit the vulnerabilities associated with loss of RB. Several groups have explicitly screened for genes/drugs that kill RB-deficient tumor cells [83,87]. Such agents could presumably be effective for RB-deficient TNBC that fail front-line chemotherapy.

## Key questions to fully leverage the retinoblastoma tumor suppressor pathway in the clinic

The preceding volumes of data provide a compelling basis to actively target the RB pathway in breast cancer. However, several key questions in particular emerge in realizing the capability of utilizing this information clinically.

### How should the retinoblastoma tumor suppressor pathway be measured/annotated?

The use of immunohistochemical staining and gene expression profiling provides an important determinant of RB pathway status in breast cancer conditions and subtypes. Clearly gene expression profiling shows a general attribute of tumor biology, while immunohistochemical analysis approaches focus on a single protein. However, what remains poorly understood is how the pathway is actually compromised in many settings. For example, while ~20% of DCIS are RB-negative and express high levels of p16ink4a, there is no indication as to how RB protein expression is lost. Current analysis of TCGA and other sequencing data suggest that RB loss must be occurring via nongenetic means. One possibility for this finding is that the expression of miR-210, which can target RB and other tumor suppressors, is associated with aggressive DCIS [88]. Similarly, many tumors with very high levels of cyclin D_1_ are not associated with gene amplification. Whether the mechanistic basis of RB-pathway deregulation is important is up for debate. However, cyclin D_1_ amplifications are generally associated with a more robust phenotype than simply overexpression of the gene. Understanding both the expression and fundamental genetic/epigenetic basis of pathway dysregulation could therefore prove clinically relevant and could yield standardized methodologies for the analysis of the RB pathway in the clinic.

### Can retinoblastoma tumor suppressor pathway status be used to direct standard treatment?

The volume of data suggesting a crucial role for the RB pathway in breast cancer therapeutic response or prognosis spans DCIS, ER-positive breast cancer, and TNBC. In each of these disease states, multiple groups have consistent data and important treatment decisions could be made. To this point, only in ER-positive breast cancer is the information used in the clinic. Deregulated RB/E2F target genes (for example, OncotypeDX) inform aggressive treatment with adjuvant chemotherapy. Similar tools could be developed in DCIS to provide disease risk assessment to help physicians choose therapeutic options. For example, the subset of TNBC tumors with low RB/E2F target genes and intact RB most probably has a very poor response to chemotherapy, and would have more benefit from other treatment options upfront, as opposed to progressing through relatively ineffective chemotherapy. The key to changing treatment requires very high levels of evidence that typically require prospective trials or retrospective/prospective analysis using clinical-grade diagnostic testing. Most laboratories simply do not have the resources to affect such change and the opportunity does not move forward, although a concerted effort in application of the RB pathway would seem relevant to inform different aspects of breast cancer management. In spite of these impediments there are active trials evaluating RB pathway status as a determinant of prognosis or therapeutic intervention (for example, ClinicalTrials.gov NCT01514565 or NCT01976169).

### Is there promise in directly targeting the retinoblastoma tumor suppressor pathway?

Underlying each one of the breast cancer subtypes are dysfunctions related to the RB pathway. The recent findings in ER-positive breast cancer demonstrate that directly intervening at the level of the RB pathway can yield large clinical dividends that are now being tackled by multiple clinical trials focused on ER-positive breast cancer (Table 1). Presumably these findings in ER-positive breast cancer could seed similar interventional strategies in Her2-positive breast cancer. Recent investigation of TNBC cases has parsed the disease into multiple subtypes. Ostensibly, within these subtypes (for example, basal) the loss of RB could represent a particularly frequent event that could be targeted by specific vulnerabilities imparted with RB loss. Conversely, within selected populations (for example, luminal AR) it may be possible to utilize CDK4/6 inhibition as an adjunct to other targeted therapies. Importantly, many of these trials targeting the RB pathway incorporate biomarkers to delineate the specific determinants of therapeutic response.

## Conclusion

In total, the hope is that the importance of the RB pathway will be utilized to inform treatment in concert with a full understanding of breast cancer biology to improve disease outcomes.

## Abbreviations

CDK: Cyclin-dependent kinase; DCIS: Ductal carcinoma *in situ*; EMT: Epithelial–mesenchymal transition; ER: Estrogen receptor; RB: Retinoblastoma tumor suppressor; TNBC: Triple-negative breast cancer.

## Competing interests

The authors declare that they have no competing interests.
